# A dosimetric and radiobiological evaluation of VMAT following mastectomy for patients with left-sided breast cancer

**DOI:** 10.1186/s13014-021-01895-2

**Published:** 2021-09-06

**Authors:** Yun Zhang, Yuling Huang, Shenggou Ding, Xingxing Yuan, Yuxian Shu, Jinhui Liang, Qingfeng Mao, Chunling Jiang, Jingao Li

**Affiliations:** 1 Department of Radiation Oncology, Jiangxi Cancer Hospital of Nanchang University, Nanchang, Jiangxi 330029 People’s Republic of China; 2 Medical College of Nanchang University, Nanchang, Jangxi 330031 People’s Republic of China; 3 Key Laboratory of Personalized Diagnosis and Treatment of Nasopharyngeal Carcinoma Nanchang, Jiangxi, 330029 People’s Republic of China

**Keywords:** Left-sided breast cancer, Post-mastectomy radiotherapy, VMAT, Radiation dosimetry, Normal tissue complication probability, Secondary cancer complication probabilities, Excess absolute risk

## Abstract

**Background:**

To compare the dosimetric, normal tissue complication probability (NTCP), secondary cancer complication probabilities (SCCP), and excess absolute risk (EAR) differences of volumetric modulated arc therapy (VMAT) and intensity-modulated radiation therapy (IMRT) for left-sided breast cancer after mastectomy.

**Methods and materials:**

Thirty patients with left-sided breast cancer treated with post-mastectomy radiation therapy (PMRT) were randomly enrolled in this study. Both IMRT and VMAT treatment plans were created for each patient. Planning target volume (PTV) doses for the chest wall and internal mammary nodes, PTV1, and PTV of the supraclavicular nodes, PTV2, of 50 Gy were prescribed in 25 fractions. The plans were evaluated based on PTV1 and PTV2 coverage, homogeneity index (HI), conformity index, conformity number (CN), dose to organs at risk, NTCP, SCCP, EAR, number of monitors units, and beam delivery time.

**Results:**

VMAT resulted in more homogeneous chest wall coverage than did IMRT. The percent volume of PTV1 that received the prescribed dose of VMRT and IMRT was 95.9 ± 1.2% and 94.5 ± 1.6%, respectively (*p* < 0.001). The HI was 0.11 ± 0.01 for VMAT and 0.12 ± 0.02 for IMRT, respectively (*p* = 0.001). The VMAT plan had better conformity (CN: 0.84 ± 0.02 vs. 0.78 ± 0.04, *p* < 0.001) in PTV compared with IMRT. As opposed to IMRT plans, VMAT delivered a lower mean dose to the ipsilateral lung (11.5 Gy vs 12.6 Gy) and heart (5.2 Gy vs 6.0 Gy) and significantly reduced the V_5_, V_10_, V_20,_ V_30,_ and V_40_ of the ipsilateral lung and heart; only the differences in V_5_ of the ipsilateral lung did not reach statistical significance (*p* = 0.409). Although the volume of the ipsilateral lung and heart encompassed by the 2.5 Gy isodose line (V_2.5_) was increased by 6.7% and 7.7% (*p* < 0.001, *p* = 0.002), the NTCP was decreased by 0.8% and 0.6%, and SCCP and EAR were decreased by 1.9% and 0.1% for the ipsilateral lung. No significant differences were observed in the contralateral lung/breast V_2.5_, V_5,_ V_10_, V_20_, mean dose, SCCP, and EAR. Finally, VMAT reduced the number of monitor units by 31.5% and the treatment time by 71.4%, as compared with IMRT.

**Conclusions:**

Compared with IMRT, VMAT is the optimal technique for PMRT patients with left-sided breast cancer due to better target coverage, a lower dose delivered, NTCP, SCCP, and EAR to the ipsilateral lung and heart, similar doses delivered to the contralateral lung and breast, fewer monitor units and a shorter delivery time.

## Background

Breast cancer is the most common cancer among women. Although breast-conserving surgery for early breast cancer has become the standard treatment in European and American countries [1, 2], radical post-mastectomy remains the most accepted surgical modality in many countries, such as developing areas on the mainland of China [3]. Adjuvant post-mastectomy radiotherapy (PMRT) has been shown to effectively reduce locoregional failure and mortality in breast cancer [4, 5]. However, PMRT often involves regional lymph nodes, including for instance internal mammary nodes (IMN) and supraclavicular nodes (SCN). The covering of these lymph node regions often results in larger irradiation fields and volumes. Accordingly, organs at risk (OARs) must receive a considerable radiation dose, which increases the risk of acute and late toxicity, especially the risk of ischemic heart disease [6]. Therefore, it is especially important to adopt an appropriate radiotherapy technology (RT) that not only ensures sufficient irradiation dose coverage to the target area but also reduces the dose to the surrounding normal tissue as much as possible.

Electron beam or arc irradiation [7, 8] and three-dimensional conventional or conformal radiotherapy (3D-CRT) with tangential fields [9, 10] have been used in PMRT for more than 20 years. However, for patients with left-sided breast cancer, if IMN is involved, it is a dosimetric challenge to deliver a uniform target dose with those techniques. To achieve better target dose homogeneity and conformity results and decrease toxicity to normal tissues, intensity-modulated radiation therapy (IMRT) has been widely implemented in the clinic [11–14]. Nevertheless, dose homogeneity and the degree of conformity influenced by the target motion are increased with the addition of treatment time for IMRT [15], and the patient’s satisfaction and clinical effect also decrease due to the prolonged treatment time.

In recent years, volumetric-modulated arc therapy (VMAT) has been applied in PMRT to improve treatment efficiency [16–26]. Almost all results thus far have confirmed that VMAT achieves target coverage similar to or better than that of IMRT, although most studies evaluated the target of the chest wall (CW), IMN and SCN as the whole planning target volume (PTV). As we know, the PTV of CW and IMN usually has lower target coverage; therefore, it is necessary to evaluate it separately. For the OARs, different results have been reported [16–25]. As a result of studies by Popescu [16], Zhang [17], Zhao [18] and Hu [19], VMAT decreases the dose to the ipsilateral lung, heart and contralateral breast/lung, relative to IMRT.. However, the research of Ma [20], Xie [21] and Wang [22] demonstrates that IMRT has dosimetric advantages in the heart and left lung, compared with VMAT plans. Furthermore, the mean dose to the heart of VMAT plans in these studies is still relatively high (> 7.8 Gy)[16–26], even up to 15.2 Gy [24]. According to Darby [6], the rates of major coronary events increase linearly with the mean dose to the heart by 7.4% per gray; therefore, it is important to further reduce the radiation dose to the heart. In addition, VMAT increases low-dose radiation to large volumes of normal tissues, which will potentially enhance the estimated risk of secondary tumor and radiation-induced pulmonary and cardiac toxicity [27–29]. Furthermore, radiobiological metrics such as the normal tissue complication probability (NTCP) of pneumonitis, secondary cancer complication probabilities (SCCP) and excess absolute risk (EAR) can be evaluated and compared and can provide a more robust comparison of different radiotherapy techniques [21,-26,30–31]. However, there are fewer related studies on PMRT.

In this study, we used SmartArc optimization algorithms, Equivalent Uniform Dose (EUD) optimization parameters and virtual block to restrict the low-dose area and explored the balance between the dose to the target area and that to normal tissue in VMAT planning. We then discussed dosimetric characteristics of the targets of CW and IMN and the target of SCN, as well as the dose to the ipsilateral lung, heart, contralateral breast/lung in IMRT and VMAT plans of left-sided breast cancer PMRT. We further predicted treatment outcomes focused on the lower irradiation dose of OARs (NTCP, SCCP and EAR of the ipsilateral lung, heart and contralateral breast/lung).

## Methods and materials

### Patients and target delineation

Thirty left-sided breast cancer patients after radical post-mastectomy with a clinical-stage above T3 or N1-3 (average age: 47 years, range 25–65 years) were randomly enrolled in this study. All patients were positioned supine on a commercially available breast tilt board to render the sternum parallel to the table, with both arms fully abducted (90° or greater) and externally rotated, and the head was secured. A planning CT scan at 5-mm intervals from the mastoid process to 3 cm below the right breast fold was obtained for each patient with a CT simulator (Siemens Medical Systems).

The CTV (clinical target volume) was delineated according to the breast cancer atlas for radiation therapy planning consensus definitions of the Radiation Therapy Oncology Group (available at http://www.rtog.org/CoreLab/ContouringAtlases/BreastCancerAtlas.aspx). CTV1 was defined as the ipsilateral chest wall (CW) and IMN regions, and the SCN region was regarded as CTV2. PTVs were obtained from CTVs by expanding a 5-mm margin in three dimensions. An additional bolus was applied on PTV1 only, and PTV2 was 4 mm from the skin surface, excluding the build-up region. Mean volumes and standard deviation of PTV1, PTV2 and whole PTV were 367.19 ± 84.08 cm^3^ (range 238.19–537.88 cm^3^), 163.48 ± 31.09 cm^3^ (range 112.00–256.44 cm^3^) and 530.67 ± 98.02 cm^3^ (range 376.94–757.45 cm^3^), respectively. OARs, such as the ipsilateral lung, heart, contralateral breast and lung, liver, spinal cord and trachea, were outlined on the axial CT sections.

### Treatment planning

**Fig. 1:**
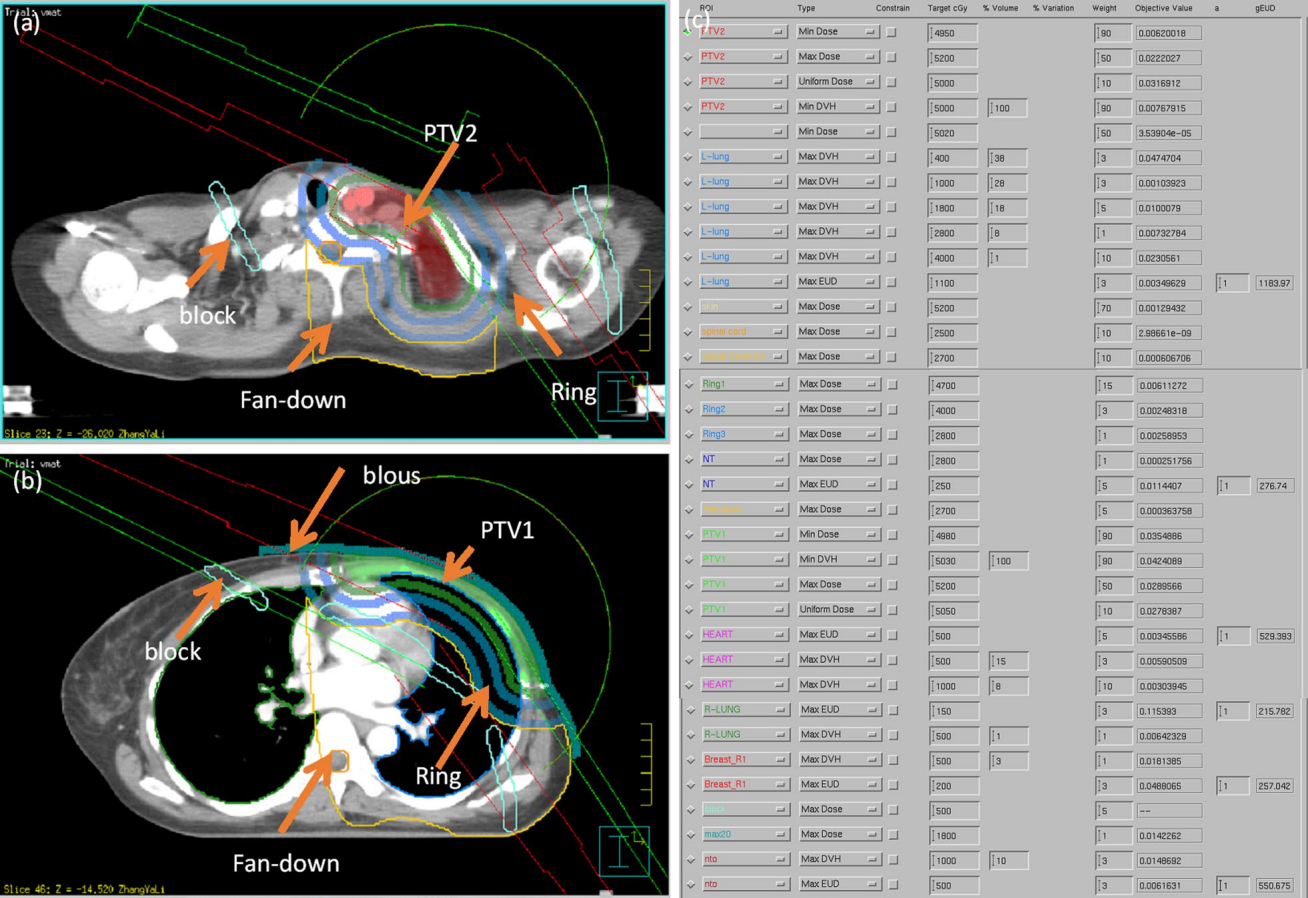
The auxiliary structure (**a**, **b**) and optimization parameters (**c**) of VMAT planning

For each patient, VMAT as well as step-and-shoot IMRT plans were created using a three-dimensional treatment planning system (Philips Pinnacle version 9.8). A Varian TrueBeam linear accelerator with a 6-MV photon energy beam was used. SmartArc and DMPO optimization types were used to optimize the VMAT and IMRT plans, respectively, and dose calculation was performed with a collapsed cone convolution algorithm. To ensure sufficient skin dose coverage, a virtual 5-mm bolus was applied to the CW of each patient before optimization and dose calculation. VMAT treatment plans were generated by two partial arcs starting from 295° to 145° clockwise and inverse, with one control point every 4°. To reduce the low dose to the normal organs, EUD and an artificially drawn block were used to limit the lower dose during plan optimization. Seven beams were used in the IMRT plan. The prescription dose was 50 Gy in 25 fractions, and the 95% PTV received 50 Gy. The aim, starting objective and constraints of planning optimization for the VMAT and IMRT plans are specified in Table 1, and the screenshot of planning tools and setting are shown Fig. 1. To objectively distinguish the dosimetric and radiobiological metrics differences between VMAT and IMRT techniques and not the respective expertise of the planner or settings of the optimizer, all plans were completed by the same planner, the same initial optimization parameters were set to the two technologies and the optimization parameters were adjusted according to the dosimetric results and objective value.

**Table 1:** Planning parameter, weight, and aims for VMAT and IMRT optimization

Structures	Type	Dose (Gy)	Volume (%)	Weight	Aim
PTV1	Min dose	49.8	–	90	V_50_ Gy ≥ 95%, V_55_ Gy ≤ 1%
	Min DVH	50.3	100	90	
	Max dose	52.0	–	50	
	Uniform dose	50.5	–	10	
PTV2	Min dose	49.5	–	90	
	Min DVH	50.0	100	90	
	Max dose	52.0	–	50	
	Uniform dose	50.0	–	10	
Ipsilateral lung	Max DVH	4.0	38	3	V_5_ Gy ≤ 50%, V_20_ Gy ≤ 25% Mean dose < 15 Gy
	Max DVH	10.0	28	3	
	Max DVH	18.0	18	5	
	Max DVH	28.0	8	3	
	Max DVH	40.0	1	10	
	Max EUD (a = 1)	11.0	3	3	
Heart	Max DVH	5.0	15	3	V_30_Gy ≤ 5% Mean dose ≤ 8 Gy
	Max DVH	10.0	8	10	
	Max EUD (a = 1)	5.0	–	5	
Contralateral lung	Max DVH	5.0	1	1	V_5_ Gy < 5%\ Mean dose ≤ 3 Gy
	Max EUD (a = 1)	1.5	–	3	
Contralateral breast	Max DVH	5.0	3	1	Mean dose ≤ 3 Gy
	Max EUD (a = 1)	2.0	–	3	
Spinal cord	Max Dose	25.0	–	10	D_max_ ≤ 30 Gy

### Plan evaluation and statistical tools

For dosimetric analysis, the following indices extracted from dose-volume histograms (DVHs) were used: (1) D_2%_, D_98%_, (dose received by 2% and 98% of PTV, near-maximum dose and minimum dose), D_mean_ (mean dose of PTV), V_95%_, V_100%_, V_107%_ and V_110%_ (percent volume receiving greater than 95% to 110% of the prescribed dose) and the dose homogeneity index (HI), conformity index (CI) and conformity number (CN) for PTV. The HI was calculated according to ICRU 83 [32]: HI = (D_2%_–D_98%_)/D_50%_. CI and CN were proposed by Paddick et al. [33]. CI = V_50_/VPTV and CN = (VPTV_50_/VPTV)*(VPTV_50_/V_50_), where VPTV is the target volume, V_50_ is the volume of prescribed isodose value and VPTV_50_ is the volume of the target that is covered by the prescribed isodose value. Smaller CI and larger CN represent better dose conformity. (2) For OARs, the mean doses and a set of appropriate V_x(Gy)_ values to the ipsilateral lung, heart, contralateral breast and lung and normal tissue were analyzed.

The NTCP for radiation-induced pneumonitis was computed for the lung using the Lyman-Kutcher-Berman model using the following values: D50 = 30.80 Gy, n = 0.98 and m = 0.37 [30]. NTCP for radiation-induced mortality for the heart was calculated using the relative seriality model using the following values: D50 = 52.4 Gy, s = 1.0 and γ = 1.3 [34]. The EAR was calculated using the following equations: β ∗ exp(γ_*e*_ (age_x_ − 30) + γ_*a*_) [31], where β is a dose–response initial slope (β_*Lung*_ = 7.5, β_*Breast*_ = 9.2), the age modifying factors γ*e* and γ*a* (γ*e*, *Lung* = 0.002, γ*a*, *Lung* = 4.23, γ*e*, *Breast* =  − 0.037, γ*a*, *Breast* = 1.7) were taken from Schneider et al*.* [31]*.* The age_x_ is the patient’s age at the time of radiation therapy, and the age_a_ is the attained age. An attained age of 75 yrs was used for cancer risk assessments. OED is organ equivalent dose and was calculated using the linear model for the contralateral breast, ipsilateral and contralateral lungs, and whole lungs [35] *V*_*t*_ is the total organ volume, and *V*_*i*_ is the volume receiving dose *D*_*i*_. SCCP was calculated using the product of the OED and the organ-specific absolute cancer incidence rate in percent per gray *(In*_org_) [36], and the *In*_org_ for the lungs and contralateral breast were 1.68% Gy^−1^ and 0.78% Gy^−1^ from a previous publication [37].

Statistical analyses were performed to compare the two different techniques using a paired t-test, a *P*-value ≤ 0.05 was considered the threshold for statistical significance.

## Results

### Target coverage and homogeneity

**Fig. 2:**
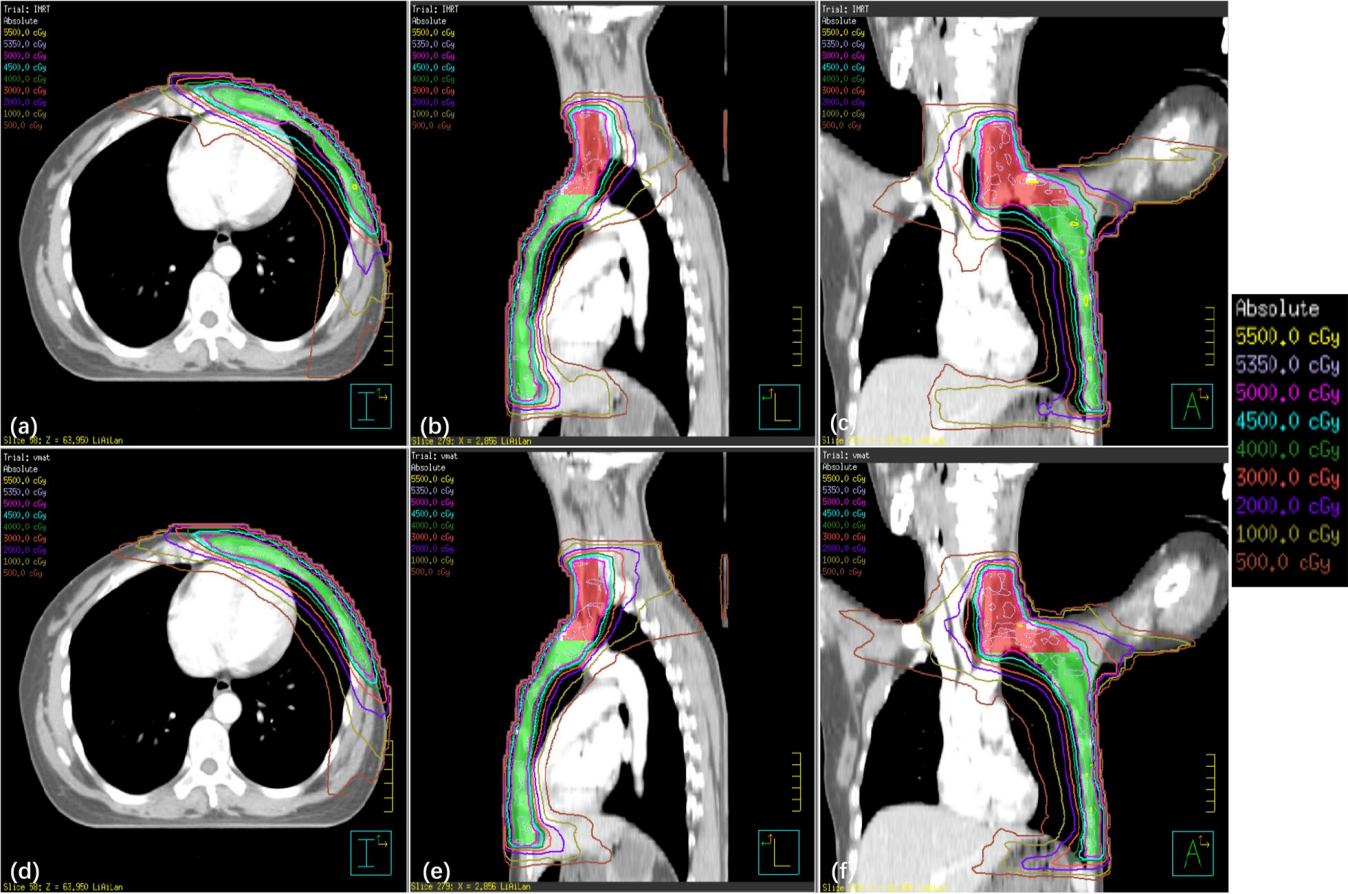
A direct comparison of dose distribution between IMRT (**a**–**c**) and VMAT (**d**–**f**). The doses represented by the different colored lines are marked on the right side of the graph

**Fig. 3:**
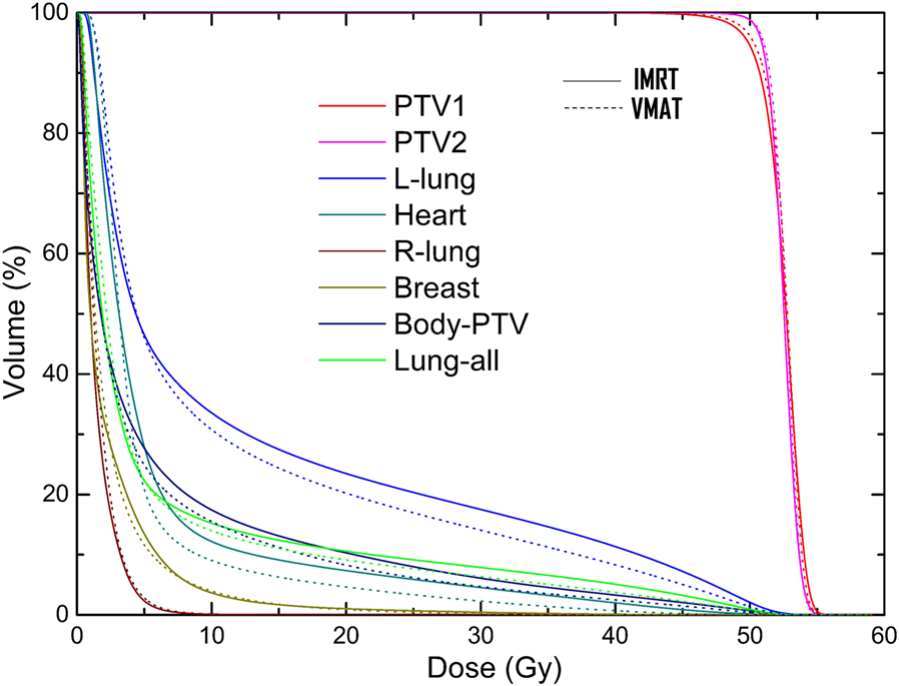
Average dose-volume histogram (DVH) comparison for PTVs and OARs with IMRT and VMAT plans. (Solid line is IMRT and dot line is VMAT)

Figure 2 shows one patient’s transversal, coronal and sagittal dose distributions. The average dose-volume histogram (DVH) of 30 patients for PTVs and OARs with IMRT and VMAT plans are shown in Fig. 3. More dosimetric parameters of PTV1, PTV2 and PTV of IMRT and VMAT plans with all patients are presented in Table 2. It was evident that the VMAT plan provided better coverage of PTV. For PTV2, no substantial differences were observed between IMRT and VMAT, while VMAT plans showed superiority compared with IMRT in PTV1 in most dosimetric parameters, except the parameters of V_107%_. The average percent volume of PTV1 receiving the prescribed dose for IMRT and VMAT was 94.5 ± 1.6% and 95.9 ± 1.2% (*p* < 0.001), that receiving 110% of the prescription dose was 0.3 ± 0.2% for IMRT and 0.2 ± 0.1% for VMAT (*p* = 0.018), the near-maximum dose and minimum dose were 54.5 ± 0.3 Gy and 48.3 ± 0.6 Gy for IMRT and 54.3 ± 0.2 Gy and 48.7 ± 0.3 Gy for VMAT (*p* = 0.011, 0.003) and the HI was 0.12 ± 0.02 for IMRT and 0.11 ± 0.01 for VMAT (*p* = 0.001). For the whole PTV, VMAT plans showed superiority over IMRT with respect to conformity, in which the average CI and CN were 1.10 and 0.84 for the VMAT plans and 1.17 and 0.78 for the IMRT plans, respectively.

**Table 2:** PTV dose parameters of IMRT and VMAT

	Parameters	IMRT	VMAT	P-value
PTV1	V_95%_ (%)	99.0 ± 0.5	99.3 ± 0.3	0.001
	V_100%_ (%)	94.5 ± 1.6	95.9 ± 1.2	< 0.001
	V_107%_ (%)	22.1 ± 7.9	20.9 ± 8.2	0.548
	V_110%_ (%)	0.3 ± 0.2	0.2 ± 0.1	0.018
	D_2%_ (Gy)	54.5 ± 0.3	54.3 ± 0.2	0.011
	D_98%_ (Gy)	48.3 ± 0.6	48.7 ± 0.3	0.003
	D_mean_ (Gy)	52.4 ± 0.2	52.6 ± 0.2	0.014
	HI	0.12 ± 0.02	0.11 ± 0.01	0.001
PTV2	V_95%_ (%)	99.9 ± 0.1	99.9 ± 0.1	0.374
	V_100%_ (%)	98.8 ± 0.6	99.0 ± 0.6	0.173
	V_107%_ (%)	13.6 ± 7.3	15.5 ± 9.8	0.264
	V_110%_ (%)	0.1 ± 0.1	0.1 ± 0.1	0.638
	D_2%_ (Gy)	54.1 ± 0.4	54.0 ± 0.4	0.233
	D_98%_ (Gy)	50.2 ± 0.3	50.3 ± 0.4	0.207
	D_mean_ (Gy)	52.5 ± 0.3	52.6 ± 0.4	0.055
	HI	0.08 ± 0.01	0.07 ± 0.01	0.075
PTV	CI	1.17 ± 0.05	1.10 ± 0.03	< 0.001
	CN	0.78 ± 0.04	0.84 ± 0.02	< 0.001

### Dose, NTCP and EAR analysis of OARs

As shown in Fig. 3, the VMAT plans provided superior ipsilateral tissue (ipsilateral lung and heart) and normal tissue (body-PTV) sparing and not only reduced the region in the medium to high doses but also had lower volumes in low-dose regions. Table 3 presents the results of DVH numerical analysis of the OARs: ipsilateral lung, heart, contralateral lung and breast and all normal tissue (body-PTV). Compared with IMRT plans, VMAT plans typically decreased V_5_, V_10_, V_20_, V_30_ and V_40_ of the ipsilateral lung and heart. Only the differences in V_5_ of the ipsilateral lung did not reach statistical significance (*p* = 0.409). Ipsilateral lungs that received 10, 20, 30 and 40 Gy were reduced by 2.8%, 3.3%, 3.4% and 3.0% in VMAT plans. Hearts that received more than 5, 10, 20, 30, and 40 Gy were reduced by 7.0%, 3.1%, 2.8%, 2.2% and 1.3% in VMAT plans, respectively. The average mean doses to the ipsilateral lung and heart were reduced by 1.1 and 0.8 Gy, respectively. Whereas VMAT would result in larger volumes of the ipsilateral lung and heart receiving low doses, the average volume percentage receiving the 2.5 Gy dose increased by 6.7% and 7.7%, respectively (*p* < 0.001, *p* = 0.002). Moreover, the V_5_, V_20_ and D_mean_ of body-PTV decreased in VMAT plans. For the contralateral lung and breast, the differences between VMAT and IMRT plans did not reach statistical significance.

**Table 3:** Dose comparison of OARs of VMAT and IMRT

Structure	Parameters	IMRT	VMAT	VMAT-IMRT	*P*
Ipsilateral lung	V_2.5_ (%)	68.7 ± 8.1	75.4 ± 7.0	6.7	< 0.001
	V_5_ (%)	46.5 ± 3.2	46.1 ± 2.9	− 0.4	0.409
	V_10_ (%)	33.6 ± 1.7	30.8 ± 1.8	− 2.8	< 0.001
	V_20_ (%)	23.5 ± 1.7	20.2 ± 1.5	− 3.3	< 0.001
	V_30_ (%)	17.5 ± 1.4	14.1 ± 1.3	− 3.4	< 0.001
	V_40_ (%)	11.3 ± 1.3	8.3 ± 1.2	− 3.0	< 0.001
	D_mean_ (Gy)	12.6 ± 0.7	11.5 ± 0.7	− 1.1	< 0.001
Heart	V_2.5_ (%)	61.0 ± 14.1	68.7 ± 11.6	7.7	0.002
	V_5_ (%)	27.9 ± 7.9	20.9 ± 7.8	− 7.0	< 0.001
	V_10_ (%)	12.2 ± 3.8	9.1 ± 3.4	− 3.1	< 0.001
	V_20_ (%)	7.4 ± 2.6	4.6 ± 1.7	− 2.8	< 0.001
	V_30_ (%)	4.5 ± 1.7	2.3 ± 1.0	− 2.2	< 0.001
	V_40_ (%)	2.0 ± 1.1	0.7 ± 0.5	− 1.3	< 0.001
	D_mean_ (Gy)	6.0 ± 1.1	5.2 ± 0.9	− 0.8	< 0.001
Contralateral breast	V_2.5_ (%)	28.1 ± 10.0	28.0 ± 11.8	− 0.1	0.987
	V_5_ (%)	13.5 ± 5.3	11.6 ± 5.2	− 1.9	0.109
	V_10_ (%)	3.7 ± 2.2	4.0 ± 2.5	0.3	0.504
	V_20_ (%)	1.0 ± 1.0	0.9 ± 0.9	− 0.1	0.179
	D_mean_ (Gy)	2.5 ± 0.6	2.5 ± 0.8	0.0	0.677
Contralateral lung	V_2.5_ (%)	16.9 ± 11.5	19.3 ± 10.0	2.4	0.199
	V_5_ (%)	2.3 ± 2.0	2.8 ± 2.7	0.5	0.114
	V_10_ (%)	0.1 ± 0.1	0.1 ± 0.3	0.0	0.709
	D_mean_ (Gy)	1.5 ± 0.5	1.6 ± 0.4	0.1	0.087
Body-PTV	V_2.5_ (%)	41.9 ± 7.4	40.7 ± 5.7	− 1.2	0.314
	V_5_ (%)	27.7 ± 4.6	24.9 ± 3.3	− 2.8	< 0.001
	V_10_ (%)	17.4 ± 2.5	15.5 ± 1.8	− 1.9	< 0.001
	V_20_ (%)	10.3 ± 1.3	8.3 ± 1.0	− 2.0	< 0.001
	V_30_ (%)	6.1 ± 0.9	4.7 ± 0.6	− 1.4	< 0.001
	V_40_ (%)	3.3 ± 0.5	2.5 ± 0.3	− 0.8	< 0.001
	D_mean_ (Gy)	6.3 ± 0.8	5.7 ± 0.6	− 0.6	< 0.001
Lung-All	V_2.5_ (%)	40.3 ± 9.3	44.6 ± 8.2	4.3	0.002
	V_5_ (%)	22.3 ± 2.4	22.4 ± 2.7	0.1	0.806
	V_10_ (%)	15.2 ± 1.1	14.0 ± 1.1	− 1.2	< 0.001
	V_20_ (%)	10.6 ± 0.9	9.1 ± 0.8	− 1.5	< 0.001
	V_30_ (%)	7.9 ± 0.7	6.4 ± 0.7	− 1.5	< 0.001
	V_40_ (%)	5.1 ± 0.6	3.7 ± 0.5	− 1.4	< 0.001
	D_mean_ (Gy)	6.5 ± 0.5	6.1 ± 0.6	− 0.4	< 0.001

The SCCP and EARs of the ipsilateral lung, whole lung, contralateral lung and breast, and the NTCP of the ipsilateral lung and heart for the investigated breast radiotherapy techniques are shown in Table 4. The EAR of the ipsilateral lung and whole lung for VMAT plans were decreased by an average of 9% and 6%, compared with that of IMRT. For the contralateral lung and breast, the differences in EAR value for VMAT and IMRT plans did not reach statistical significance. VMAT plans to exhibit the minimum NTCP values for the ipsilateral lung and the heart, compared with IMRT plans.

**Table 4:** NTCP and EAR comparison of ipsilateral lung, contralateral lung, double lung, heart, and breast of VMAT and IMRT

Structure	Parameter	IMRT(%)	VMAT(%)	VMAT/IMRT	*P*
Ipsilateral lung	NTCP	4.1 ± 0.5	3.3 ± 0.5	0.79 ± 0.05	< 0.001
	SCCP	21.1 ± 1.2	19.2 ± 1.2	0.91 ± 0.03	< 0.001
	EAR	130 ± 7.09	118.87 ± 7.21	0.91 ± 0.03	< 0.001
Lung-All	NTCP	1.32 ± 0.13	1.18 ± 0.13	0.89 ± 004	< 0.001
	SCCP	10.9 ± 0.9	10.2 ± 0.9	0.94 ± 0.04	< 0.001
	EAR	67.4 ± 5.6	63.1 ± 5.6	0.94 ± 0.04	< 0.001
Contralateral Lung	SCCP	2.5 ± 0.8	2.7 ± 0.7	1.12 ± 0.24	0.087
	EAR	15.6 ± 4.7	16.9 ± 4.4	1.12 ± 0.24	0.089
Contralateral Breast	SCCP	1.9 ± 0.5	2.0 ± 0.6	1.04 ± 0.24	0.677
	EAR	14.01 ± 4.73	14.55 ± 6.02	1.04 ± 0.24	0.399
Heart	NTCP	0.86 ± 0.50	0.34 ± 0.22	0.44 ± 0.28	< 0.001

### MU and beam delivery time

Table 5 summarizes the results for all treatment plans about the number of monitor units (MU), beam-on time (BOT), and treatment time. The total MUs for VMAT plans were decreased by an average of 31.5% compared with IMRT. BOT was similar for each technique, while the treatment time was shorter for VMAT, an average decrease of 71.4%, compared with that for IMRT.

**Table 5:** The Number of Monitor Units (MU), Beam-on Time (BOT), and Treatment time with IMRT and VMAT plans

Parameter	IMRT	VMAT	Difference	*P*
MU	893 ± 142	611 ± 54	− 282	< 0.001
Beam-on time (min)	1.49 ± 0.24	1.46 ± 0.06	− 0.03	0.440
Treatment time (min)	7.00 ± 0.62	2.00 ± 0.04	− 5.00	< 0.001

## Discussion

IMRT and VMAT can shape the dose to the concave target in the CW and IMN in breast cancer radiotherapy. In this study, we systematically compared the dosimetric parameters of two techniques, IMRT and VMAT, at our institute for 30 cases of post-mastectomy left-sided breast cancer patients. The results of our study indicated that both IMRT and VMAT provided good coverage of the target, while VMAT showed, with statistical significance, more conformity and more dose homogeneity in the target area of CW and IMN, compared with those of IMRT, by avoiding areas of under-dose, and at the same time eliminating areas of relative overdose. Our VMAT significantly reduced the near-maximum dose of the PTV of CW and IMN, which was 54.3 ± 0.2 Gy, as compared with 55.4 ± 1.7 Gy from a study by Zhang [17], and 56.64 ± 0.63 Gy from a study by Hu [19], 54.93 ± 0.87 Gy from a study by Ma [20]. Furthermore, the increase in the near-minimum doses, V_95%_ and V_100%_ were higher in the VMAT plans than in the IMRT plans: 48.7 ± 0.3 Gy, as compared with 48.5 ± 2.2 Gy from a study by Zhang [17], and 48.84 ± 0.41 Gy from a study by Hu [19] and 47.77 ± 0.35 Gy from a study by Ma [20]. The values of HI and CN in our study signify that slightly better homogeneity and conformality of target coverage were attained in VMAT plans than in IMRT plans. These results were also better than those reported by other studies [17–21, 26]. Improving the homogeneity of irradiation is vital for PMRT in locally advanced breast cancers because it may reduce the acute complication rate, as well as the occurrence of long-term fibrosis [38]. We also found that, with IMRT plans, it was difficult to achieve dose coverage for targets with CW and IMN of large curvature, while the VMAT plan was easier to achieve, in close agreement with the results of Zhang [17].

In the optimization of VMAT and IMRT, the heart, ipsilateral lung and contralateral breast were considered the three most important OARs due to their large volumes. These OARs were protected by adjusting the priority values to reduce the maximum percent dose and scatter dose. Compared with our IMRT plans and other studies of VMAT plans, the mean dose to the heart was comparably lower in our VMAT plans, 5.2 ± 0.9 Gy, while it was 13.5 ± 5.0 Gy according to Zhang [17], 7.2 ± 2.3 Gy according to Zhao [18], 9.31 ± 1.62 Gy according to Hu [19], 11.9 ± 5.06 Gy according to Ma [20], 7.7 ± 1.1 Gy according to Xie [21] and 7.4 ± 1.4 Gy according to Wang [22],15.2 ± 2.2 Gy according to Nobnop [24], 9.3 ± 1.1 Gy according to Zhang [26]. The low and medium doses received by the heart were also significantly lower with the VMAT technique than with the IMRT technique, except for V_2.5_. In our study, the volume of heart received more than 2.5 Gy (lower dose) was increased by 8.80%, whereas the value in VMAT was also clinically acceptable. In addition, our NCTP values based on the relative seriality model further verified that the VMAT plans provide better protection for the heart (0.34% vs 0.86%). This result differs from those of Wang et al. [22], in which the mean dose, V_5_, V_10_, V_20_, and V_30_ of the heart are the highest for VMAT out of these techniques. For the ipsilateral lung and whole-lung, both mean dose and volume received more than 5 Gy, 10 Gy, 20 Gy and 30 Gy were lower in VMAT plans; only the differences in the V_5_ were not statistically significant. Although the V_2.5_ was increased in VMAT plans, the risks of pulmonary toxicity, SCCP and EAR were not increased. Compared with previous studies of VMAT plans for PMRT patients [16–24], the low-dose exposure volume of the ipsilateral lung and heart were lower, and the values of the ipsilateral lung and heart V_5_ of VMAT plans were also lower, 46.1% and 20.9%. These values were 83.0% and 70.2% according to Popescu [16], 61.1% and 78.0% according to Zhang [17], 53.91% and 61.52% according to Hu [19], 70.36% and 42.33% according to Ma [20], 35.7% (whole lung) and 48.6% according to Xie [21] 48.9% and 25.5% according to Wang [22] and. 43.5% (whole lung) and 66.9% according to Zhang [26].

Except for acute and late radiation damage to the heart and ipsilateral lung, the delivery of low-dose irradiation to healthy tissue, especially to the contralateral breast and lung, has been estimated to double the risk of subsequent malignancy, and this risk is enhanced with increasing dose [27]. Based on our study, it was demonstrated that VMAT would not significantly increase the dose to the contralateral tissue compared with IMRT plans (*p* < 0.05), which is contradictory to the reports of Wang et al. [22] and can be explained by differences in field setups.The tangential fields were used in IMRT plans, allowing full avoidance of the contralateral breast and lung. On the other hand, with fully modulated multibeam IMRT techniques, such as sever beam with gantry angles of 300°, 320°, 340°, 20°, 100°, 115° and 130°, a larger volume of normal tissue is exposed to a ‘low-dose bath’. However, there are potential advantages for special anatomical situations in the presence of proximity of the heart to the inner side of the CW or when irradiation of the internal mammary lymph-node chain is indicated. And IMRT planning with two or four tangential photon beam arrangements in PMRT patients can be challenging. As reported by Zhang et al. [26], the CN of the target area of the CW was only 0.51 in 2-tangent fields IMRT plans and 0.68 in 4-tangent fields IMRT plans. In a dosimetric analysis of 85 patients using multi-fields (6–12 fields) [13], the homogeneity and conformity were improved, in which the HI and CI of PTV could reach 0.13 and 1.41.

These results show that VMAT is the optimal technique for all PMRT patients, especially considering the complexity of the target and patient geometry. In our study, to find the balance between the dose to the target area and normal tissue in VMAT plans, it is possible to reduce the low-dose radiation to normal tissue while the target coverage and conformality reach the level of IMRT plans. We adopted the following in VMAT planning. (1) EUD parameters were used to control the dose of OARs during optimization, the EUD has more advantages in controlling the mean dose of OARs [39]. (2) Virtual block was used to restrict the low-dose area, so the MU of VMAT was small when the gantry angle was vertical with the PTV of CW and IMN using virtual block, it only improved the PTV distribution without increasing the dose of OARs. (3) Optimization parameters were set for target areas separately and additional optimization parameters added to targets areas where it was difficult to reach the prescribed dose. In the above ways, our VMAT plans achieved the initial goal, the V5 of OARs was well controlled, such as < 50% in the ipsilateral lung, < 25% in the heart, < 3% in the contralateral lung, < 12% in the contralateral breast and < 25% in all normal tissues. These results are similar to those of other groups planned with tangent field IMRT [21, 26]. Lai et al. proposed [23] that the low-dose regions could be further reduced by using modified VMAT plans with the half-field technique and flattening filter-free beams, which lower the dose to the heart even lower than that in 3D-CRT. We will perform further research on this topic in the future.

Another clinical advantage of the use of VMAT is that it generally takes fewer MUs and improves the efficiency of plan delivery, compared with IMRT plans. Our results showed that the total MUs for VMAT plans were decreased by an average of 31.5%, and the treatment time was decreased by an average of 71.4%, compared with that of IMRT, consistent with other studies [17, 18].

Finally, we acknowledge that the impact of respiratory movement on the CW was not considered in this study, which is the major drawback for this work. Deep inspiration breath-hold (DIBH) during breast treatment radiation had been demonstrated to not only eliminate the effects of breathing, but also to reduce considerably the dose to the heart [40–44]. For left breast-conserving surgery without regional lymph node, Karpf [40] indicated that the mean heart dose was 4.02 Gy in combination of VMAT and DIBH. And Osman [41] showed the mean heart dose was reduced from 5.8 Gy ( free breathing) to 4.1 Gy for radiation therapy including regional lymph nodes. The lower mean heart dose (2.6 Gy) for including the internal mammary chain was achieved by Ranger in 40 Gy/15 fractions [42]. Dumane [43] found that the mean heart dose to the heart was reduced on average by 2.9 Gy (8.2 to 5.3 Gy) with the addition of DIBH to VMAT in breast cancer patients with implant reconstruction receiving regional nodal irradiation. Thereby, the German society of radiation oncology breast cancer expert panel recommends the use of DIBH as the best heart-sparing technique, and a combination of DIBH and IMRT or VMAT may be used for IMN radiotherapy [44]. Furthermore, the DIBH may also play an important role in the robustness of the VMAT treatment delivery, since it reduces the respiratory-induced movement of the target. Therefore, further research is needed to address these issues.

## Conclusions

The VMAT and IMRT techniques were evaluated for PMRT with the left-sided breast cancer patients in this study. Our dosimetric analyses demonstrate that VMAT plans confer advantages in terms of the PTV of CW dose coverage and homogeneity, reduce the mean dose and volumes both in the low- and medium-dose regions of the lung and heart and decrease MU and treatment time, as compared with IMRT plans. Based on estimated risks for OARs, VMAT was the appropriate PMRT technique for PMRT patients who are prone to developing radiogenic side effects. Overall, the VMAT plan is superior to the IMRT plan.

## Data Availability

The data used and analyzed during the current study are available from the corresponding author on reasonable request.

## Abbreviations

VMAT: Volumetric modulated arc therapy
IMRT: Intensity-modulated radiation therapy
PMRT: Post-mastectomy radiation therapy
CW: Chest wall
IMN: Internal mammary nodes
SCN: Supraclavicular nodes
PTV: Planning target volume
DVH: Dose-volume histograms
HI: Homogeneity index
CI: Conformity index
CN: Conformity number
OARs: Organs at risk
RT: Radiotherapy
3D-CRT: Three-dimensional conformal radiotherapy
EUD: Equivalent Uniform Dose
CTV: Clinical target volume
NTCP: Normal tissue complication probability
SCCP: Secondary cancer complication probabilities
EAR: Excess absolute risk
DIBH: Deep inspiration breath-hold
